# Effect of pH on Effective Slip Length and Surface Charge at Solid–Oil Interfaces of Roughness-Induced Surfaces

**DOI:** 10.3390/mi12070752

**Published:** 2021-06-26

**Authors:** Porui Tian, Yifan Li

**Affiliations:** College of Metrology and Measurement Engineering, China Jiliang University, Hangzhou 310018, China; s1802080431@cjlu.edu.cn

**Keywords:** solid–oil interface, surface charge density, pH value, boundary slip, AFM

## Abstract

**Featured Application:**

**Drag reduction in many applications of micro-/nano-fluidic channel.**

**Abstract:**

In the development of micro/nano fluid control systems, fluid resistance has always been one of the key factors restricting its development. According to previous studies, it is found that the boundary slip effect of the solid-liquid interface can effectively reduce the resistance of the microfluid and improve the transport efficiency of the microfluid. The boundary slip length is mainly affected by surface wettability, roughness, and surface charge density. Among them, the influence mechanism of surface charge density on the boundary slip is the most complicated, and there is a lack of relevant research, and further investigation is needed. In this paper, we present research on quantification of effective slip length and surface charge density, where the roughness effect is considered. The electrostatic and hydrodynamic force data obtained from atomic force microscopy (AFM) measurements were fitted and processed for comparative analysis. We obtained the variation of surface charge density and effective slip length when different oleophobic surface samples were immersed in ethylene glycol with different pH values. The effect of pH on the surface charge density and effective slip length was investigated by their variations. The mechanism of the effect of pH on the surface charge density was discussed. The experimental results show that in the ethylene glycol solution, no matter whether the pH value of the solution increases or decreases, the charge density of the surface with the same properties decreases, and the effective boundary slip length also shows a downward trend. In deionized water, the surface charge density and effective boundary slip length decreases with the decrease of PH value.

## 1. Introduction

The transportation of fluids in the micro/nanochannels of micro-/nano-fluidic devices have are wide range application in biological, chemical, and medical fields. Micro/nano flow is subjected to high drag with decreasing dimensions and large surface-to-volume ratio, and the reduction of drag is a crucial issue for the transport of micro/nano fluid [1]. Advancements in the fabrication of micro-/nano-fluidic systems rely on understanding the physical mechanisms of boundary conditions at solid–liquid interfaces. Boundary slip suggests that the relative velocity between the solid surface and the fluid flow at the interface is not zero, which is characterize ed by the so-called slip length [2], via $v=b{\frac{dv}{dy}|}_{y=0}$, where *b* is the slip length, *v* is the tangential velocity, and *y* is the axis perpendicular to the wall. It is obvious that boundary condition at the solid–liquid interface has direct influence to the hydrodynamic drag, as published studies show that boundary slip plays an important role in inhibiting hydrodynamic drag [3,4,5,6]. The slip length of various solid–liquid interfaces has been measured, and the slip lengths of the order of few nanometers to several micrometers are reported [7,8,9,10,11,12,13,14].

The surface charge is considered to be another factor that affects the resistance of microfluidics. Most solid surfaces immersed in an aqueous solution can change the charge density of the solid surface through actions such as adsorption of ions, dissociation of ionizable groups, and acid-base reactions. When the solid and liquid are in contact with each other, the solid surface absorbs a part of ions from the liquid and ionizes a part of its own ions, so that the two sides of the solid–liquid interface have ions of different polarities, forming an electric double layer (EDL) at the solid–liquid interface structure. It is precisely because of the existence of this electric double layer structure that the resistance of the microfluidic movement changes. Many phenomena at the solid–liquid interface are related to interface charges, such as boundary slip, nanobubbles, and surface wettability. These phenomena also provide the possibility for the future microfluidic resistance control. The influence of surface charge on the resistance of microfluidic channels is widely believed to exist. It can be based on the direct influence of the electric double layer structure, and can also be indirectly influenced by the boundary slip coupling [15,16,17,18,19]. Joly et al. used molecular dynamics simulation to study the relationship between surface charge and boundary slip length [20]. Rezaei et al. studied the electroosmosis effects at the interface of an aqueous NaCl solution and a charged silicon surface [21]. They reported that the effective slip length is found to be changed linearly dependent on the surface charge density. Manoel et al. derived an analytical formula to extract the saturation surface charge density and the slip length from experimental conductance measurements in nanopores [22]. Quantitatively studies of the surface charge changes when the OTS and glass surface were immersed in two salt solutions with different ion concentrations and deionized water with different pH values by Jing [13]. In his research, the interface charge density was changed by applying an electric field and changing the PH value of the solution. The final results show that the absolute value of the charge density of the solid–liquid interface is inversely proportional to the boundary slip length [23].

In previous studies, the electrolyte solution was used as the main research object. Oils with low surface tension are also commonly used in many fluid flow applications. Micro/nano channels use special oleophobic surfaces to guide low surface tension liquids. The roughness of oleophobic and super-oleophobic surfaces is greater than that of lipophilic surfaces. However, previous studies have focused on smooth surfaces, and have not studied the relationship between the effective slip length of rough surfaces and the surface charge density. Considering the problem of roughness, the effective boundary slip measurement model is different from the smooth surface measurement model. It is necessary to set a suitable reference surface and perform additional calculations. In addition, due to the dielectric properties of these oils, there are no research reports detailing the effect of surface charge on the effective slip length of the sample immersed in the oil.

In this paper, considering the roughness of the sample, the effect of surface charge density on the effective boundary slip when super-oleophilic, oleophobic and super-hydrophobic surfaces are immersed in ethylene glycol with different pH values is studied and analyzed. The surface charge density was varied by changing the pH of the glycol solution. The electrostatic and hydrodynamic force data between the probe and the sample were obtained using atomic force microscopy (AFM) measurements. After fitting the measured data, the surface charge density and effective boundary slip length are obtained by calculation. At the same time, the mechanism of the influence of the pH value of the solution on the surface charge density is discussed. Combining the surface charge density and effective boundary slip length measurements, we found that the surface charge density is closely related to the effective boundary slip. Due to the wide application of ethylene glycol with low surface tension, we chose to be the liquid study subject, and deionized (DI) water was chosen as the comparison reference.

## 2. Experimental

### 2.1. Experimaltal Setup 

**Figure 1:**
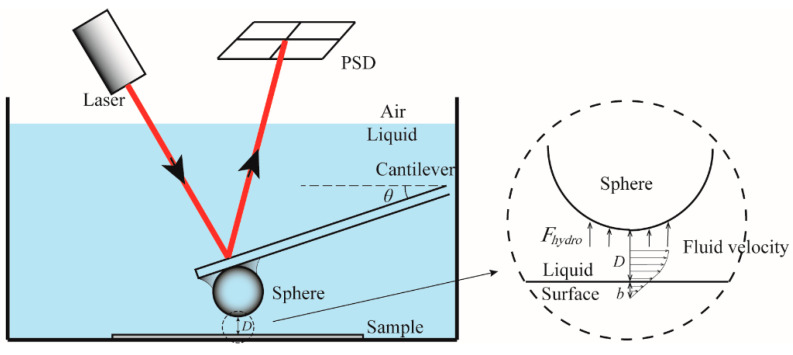
Schematic diagram of measurement of AFM colloidal probe in contact mode. The arrows in the cross-sectional view of the solid–liquid interface represent the size and direction of the fluid flow.

An AFM (D3000, Bruker, Billerica, MA, USA) operated in contact mode under liquid was used to measure the hydrodynamic force and the electrostatic force [23]. In this experiment, a colloidal probe sphere was used for measurement. First, a borosilicate sphere (GL018B/45-33, MO-Sci Corporation, Rolla, MO, USA) is glued to the end of the AFM probe (ORC 8, Bruker, Billerica, MA, USA) with epoxy resin (LG-330, Araldite, Shanghai, China). The diameter of the small sphere is about 57.6 μm. As shown in Figure 1, the colloidal probe with borosilicate sphere was driven toward the surface immersed in the liquid at a certain driving velocity. Then, the force applied on the sphere as a function of deflection was obtained by detecting the laser reflection of cantilever. The measured deflection data of the probe was used to calculate the hydrodynamic force data using Hooke’s law. For a flat surface with non-slip, the deflection can be expressed as [24,25,26],

(1) $$Def=\frac{F_{hydro}}{k}=\frac{6\pi \eta R^{2}}{kD}V,$$

where *F_hydro_* is the hydrodynamic force, *k* is the stiffness of the cantilever, *Def* is the deflection of the cantilever, *η* is the dynamic viscosity of the liquid, *R* is radius of the borosilicate sphere, *D* is the separation distance between the bottom of sphere and the surface, and *V* is the approaching velocity of the sphere. For a flat surface at boundary slip condition, the hydrodynamic force can be express as,

(2) $$\frac{V}{F_{hydro}}=\frac{1}{6\pi \eta R^{2}}\left(D+b\right),$$

where *b* is the slip length. Fit the *V/F_hydro_* curve and extend it to the *X*-axis (separation distance). At this time, the intercept of the curve on the *X*-axis (separation distance) is the boundary slip length.

During the measurement of the boundary slip length, the speed of the probe approaching the sample surface is 38.5 μm/s. The force on the probe mainly includes hydrodynamic force, electrostatic force, van der Waals force (van der Waals force can be ignored). When the approach speed of the probe decreases, the hydrodynamic force decreases, while the electrostatic force remains unchanged. Surface charge density measurement, a low velocity of 0.22 µm/s was used. The hydrodynamic force on the probe is less than 0.1 nN and can be neglected. Thus, the measured force can be considered as electrostatic force, because the hydrodynamic force at low velocity is negligible as compared to the electrostatic force. The electrostatic force data is used to obtain the surface charge density according to the modeling to be presented later. Therefore, the force data (including hydrodynamic force and electrostatic force) measured at the higher approach velocity of the probe minus the force data (mainly electrostatic force) at the lower approach velocity can obtain the hydrodynamic force, and then get the boundary slip length.

### 2.2. Quantitation of Surface Charge Density and Effective Slip Length on the Rough Surface

#### 2.2.1. Surface Charge Density

The surface charge density at the solid–liquid interface cannot be directly measured. According to previous studies, the surface charge density is closely related to the ion concentration in the liquid environment and its ion Debye length. In addition, there are various differences on the surface of different properties. A method to calculate the charge density on the surface of glass and silica in contact with an aqueous electrolyte is proposed [27]. Charge regulation of silica was found to be effective when separations well beyond Debye lengths in common solution conditions. It also proved to be very sensitive to the chemical properties of relative surfaces. Dove et al. conducted a study on the surface charge density of silica in alkaline metal chloride solutions [28]. Alex et al. used particle-based Brownian dynamics simulation tools to characterize ion groups in nanopores and simulate the conduction through nanopores at various bulk electrolyte concentrations to calculate the surface charge density of silica [29].

**Figure 2:**
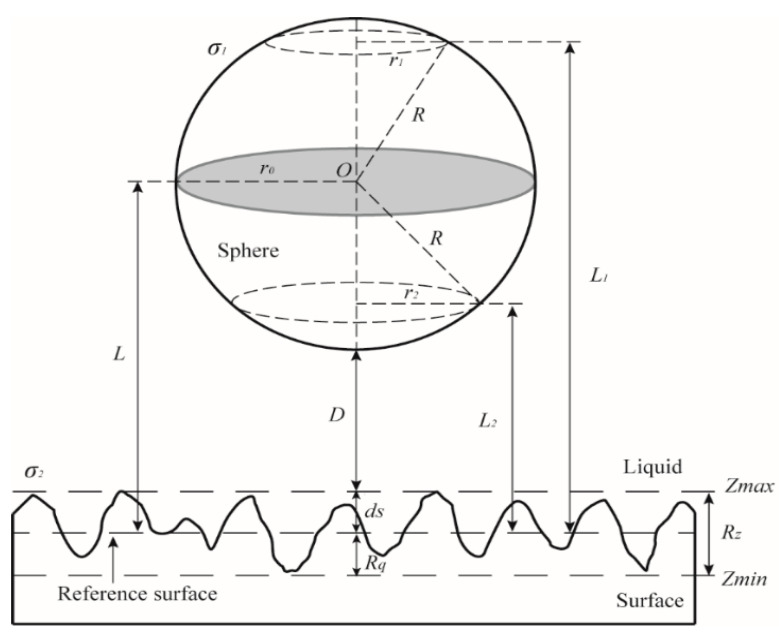
Schematic illustration of the charged sphere-rough surface system with electrostatic force between the sphere and the surface.

In this paper, the surface charge density is calculated indirectly by using AFM to measure the interface electrostatic force. For rough surfaces, the electrostatic force applied on the sphere is investigated to obtain surface charge density. The AFM experimental system can be simplified to be a sphere-rough surface system, as shown in Figure 2. We assume that the system is charged. Consider a sphere having surface charge density *σ*_1_ and a particle rough surface having surface charge density *σ*_2_, where the surface charge densities are constant and uniform. According Coulomb’s law, the electrostatic force between an arbitrary point at given horizontal section of the sphere and the surface can be expressed as [30]:

(3) $$F\left(L\right)=\frac{2}{\varepsilon \varepsilon_{0}}\left[\frac{\left(\sigma_{1}^{2}+\sigma_{2}^{2}\right)+\sigma_{1}\sigma_{2}\left(e^{\kappa L}+e^{-\kappa L}\right)}{{\left(e^{\kappa L}-e^{-\kappa L}\right)}^{2}}\right],$$

where *L* is the separation distance between an arbitrary horizontal section of the sphere and the reference surface, *ε* is the relative permittivity of the fluid, *ε*_0_ = 8.85 × 10^−12^ is the vacuum permittivity, *e* is the elementary charge, and *κ*^−1^ is the Debye length of the liquid, which is given by:

(4) $$\kappa^{-1}=\sqrt{\frac{\varepsilon \varepsilon_{0}k_{0}T}{2n_{0}e^{2}z^{2}}},$$

where *k*_0_ = 1.38 × 10^−23^ is the Boltzmann constant [31], *T* is the temperature, *n*_0_ is the original ionic concentration of the liquid, *e* is the elementary charge, and *z* is the valence number of ions. Thus, the total electrostatic force *F_electro_* applied on the sphere can be derived by integrating the Equation (1) along the vertical axis as,

(5) $$F_{electro}\left(L\right)=\int_{D+ds+2R}^{D+ds+2R}F\left(L\right)2\pi r_{0}dr_{0},$$

where *D* is the separation distance between the bottom of sphere and the peak of surface, *d_s_* is the distance between the reference surface and the peak of surface, *R* is the radius of the sphere, and *r*_0_ is the radius of the given horizontal section of the sphere.

The amplitude parameters of rough surface are the peak to valley roughness (*R_z_*), and the root mean squared (RMS) value *R_q_*. Rz is defined as the distance between the highest value of vertical position on the surface, i.e., the peak (*Z_max_*), and the lowest value of vertical position on the surface, the valley (*Z_min_*), as indicated by *Rz* = *Z_max_* − *Z_min_*. The reference surface is the surface from which the separation distance between the sphere and the rough surface is determined.

In order to obtain the electrostatic force, the sphere is divided into an upper section and a lower section to obtain the differential of *r*_0_ in Equation (5). Assume the given horizontal section of the upper and lower hemisphere having radius of *d*_1_ and *d*_2_, separately. The relationship between the radius and the vertical distance can be expressed as:

(6) $$\{\begin{array}{c} \sqrt{R^{2}-r_{1}^{2}}=D+R_{z}-R_{q}+R-L_{1}\\ \sqrt{R^{2}-r_{2}^{2}}=D+R_{z}-R_{q}+R-L_{2} \end{array},$$

where *L*_1_ is the distance between a given horizontal section of the upper hemisphere, *L*_2_ is the distance between a given horizontal section of the lower hemisphere. By taking the derivative with respect to *r*_1_ and *r*_2_ for fixed *D*, the differential of *r*_0_ can be express as:

(7) $$\{\begin{array}{c} r_{1}dr_{1}=\sqrt{R^{2}-r_{1}^{2}}dL_{1}=\left(R+D+R_{z}-R_{q}-L_{1}\right)dL_{1}\\ r_{2}dr_{2}=\sqrt{R^{2}-r_{2}^{2}}dL_{2}=\left(R+D+R_{z}-R_{q}-L_{2}\right)dL_{2} \end{array},$$

The solution of Equation (5) considering the Equations (2) and (7) leads to an expression about the electrostatic force applied on the upper section and the lower section of sphere respectively as:

(8) $$\begin{array}{ll} {\left(F_{electro}\left(D\right)\right)}_{1} & =\int_{D+R_{z}-R_{q}}^{D+R_{z}-R_{q}+2R}F\left(L_{1}\right)2\pi r_{1}dr_{1}\\ & =\int_{D+R_{z}-R_{q}}^{D+R_{z}-R_{q}+2R}F\left(L_{1}\right)2\pi r_{1}\left(R+D+R_{z}-R_{q}-L_{1}\right)dL_{1} \end{array}$$

(9) $$\begin{array}{ll} {\left(F_{electro}\left(D\right)\right)}_{2} & =\int_{D+R_{z}-R_{q}}^{D+R_{z}-R_{q}+2R}F\left(L_{2}\right)2\pi r_{2}dr_{2}\\ & =\int_{D+R_{z}-R_{q}}^{D+R_{z}-R_{q}+2R}F\left(L_{2}\right)2\pi r_{2}\left(R+D+R_{z}-R_{q}-L_{1}\right)dL_{2} \end{array}$$

Thus, the total electrostatic force between the sphere and the sample can be expressed by combining Equations (8) and (9) as:

(10) $$\begin{array}{ll} F_{electro}(D) & ={\left(F_{electro}(D)\right)}_{1}+{\left(F_{electro}(D)\right)}_{2}\\ & =\frac{4\pi \sigma_{1}\sigma_{2}}{\varepsilon_{0}\varepsilon \kappa }[\frac{R}{e^{\kappa (D+2R+R_{z}-R_{q})}-e^{-\kappa (D+2R+R_{z}-R_{q})}}+\frac{R}{e^{\kappa (D+R_{z}-R_{q})}-e^{-\kappa (D+R_{z}-R_{q})}}]\\ & -\frac{2\pi \sigma_{1}\sigma_{2}}{\varepsilon_{0}\varepsilon \kappa^{2}}\ln[\frac{1-e^{-\kappa (D+2R+R_{z}-R_{q})}}{1+e^{-\kappa (D+2R+R_{z}-R_{q})}}\cdot \frac{e^{\kappa (D+R_{z}-R_{q})}+1}{e^{\kappa (D+R_{z}-R_{q})}-1}]\\ & +\frac{2\pi (\sigma_{1}^{2}+\sigma_{2}^{2})}{\varepsilon_{0}\varepsilon \kappa }[\frac{R}{e^{2\kappa (D+2R+R_{z}-R_{q})}-1}+\frac{R}{e^{2\kappa (D+R_{z}-R_{q})}-1}]\\ & -\frac{\pi (\sigma_{1}^{2}+\sigma_{2}^{2})}{\varepsilon_{0}\varepsilon \kappa }\ln[\frac{e^{-2\kappa (D+2R+R_{z}-R_{q})}-1}{e^{-\kappa (D+R_{z}-R_{q})}-1}] \end{array}$$

Equation (10) shows the relationship between the electrostatic force applied on the sphere and the surface charge density, where the surface charge density of sphere *σ*_1_ and the surface charge density of the rough surface *σ*_2_ are unknown values. 

We assume that both the sphere and the surface are flat and made of borosilicate. Due to the same material, the charges of the probe and surface are considered equivalent, i.e., *σ*_1_ = *σ*_2_, while the separation distance is larger than 1/10 of Debye length. Equation (3) can be simplified and written as:

(11) $$\sigma_{1}{}^{2}=\frac{F(L)e^{-\kappa L}\varepsilon \varepsilon_{0}\left(e^{2\kappa L}-2e^{\kappa L}+1\right)}{2}$$

(12) $$\sigma_{1}=\left|\frac{\sqrt{2}}{2}\sqrt{F(L)e^{-\kappa L}\varepsilon \varepsilon_{0}}\times \left(1-e^{\kappa L}\right)\right|$$

The surface charge density of the rough surface *σ*_2_ can be obtained by substituting Equation (12) into Equation (9) as:

(13) $$\sigma_{2}=\left|\frac{1}{2}e^{-2\kappa L}\left[-e^{\kappa L}\sigma_{1}\left(e^{2\kappa L}+1\right)+\sqrt{e^{2\kappa L}\left(2F_{electro}(D)\varepsilon \varepsilon_{0}+\sigma_{1}{}^{2}\right)}\left(e^{2\kappa L}-1\right)\right]\right|$$

Equation (13) can be used to fit experimental AFM data for the measured electrostatic force as a function of separation distance to obtain the surface charge density. It should be noted that only the absolute value of surface charge density is provided here.

#### 2.2.2. Effective Slip Length

For the technique using hydrodynamic forces to derive slip length on a rough surface, the measured slip length should be replaced by the effective slip length because of roughness effect. The positions of the reference surface the surface from which slip velocity is occurred should be addressed. 

For rough surfaces, drainage of thin film between sphere and rough surface is investigated to obtain effective slip length. Consider the sphere-rough surface system as was shown in Figure 2 immersed in the Newtonian liquid. We assume that the particles are sufficiently rigid that any deformation due to hydrodynamic pressure is negligible. The distance *D* between two particles is assumed to be much less than radius *R* of sphere (*D* << *R*). The sphere approaches the surface along the vertical axis with a velocity *v* (the Reynolds problem). In order to characterize the state of the fluid flow, the boundary conditions of solid–liquid interface of two surfaces are set as Navier slip condition. By solving the motion equation, the hydrodynamic force applied on the sphere can be expressed as [32]:

(14) $$F_{hydro}=\frac{6\pi \mu R^{2}v}{\left(D+d_{s}+b_{2}+b_{eff}\right)}$$

where *b*_2_ is the slip length of the sphere, *b_eff_* is the effective slip length of the rough surface.

It should be noted that the effective slip length is obtained by investigating the velocity of liquid flow at the reference surface. Therefore, the velocity at the reference surface should be representative of the average velocity of liquid flow between the peaks and valleys of the rough surface. It is obvious that the velocity of liquid flow depends on the topography. Thus, the *R_q_* roughness of the surface profile is a typical case to describe surface roughness based on the standard of International Organization for Standardization (ISO), and is suitable to describe the average velocity of liquid flow. 

Therefore, the reference surface is defined as the position of *R_q_* roughness, and the effective slip length is defined by *b_eff_* = *b* − *b*_2_ − *d*_s_, via *ds* = *R_z_* − *R_q_*. Note that the slip length of the sphere *b*_2_ can be obtained by measuring the hydrodynamic force between the borosilicate glass sphere and borosilicate glass sample. In this case, it can be found that *b*_2_ = *b*, and slip length of sphere *b*_2_ is obtained. 

### 2.3. Preparation of Liquids and Surfaces 

#### 2.3.1. Liquids

In this experiment, ethylene glycol and deionized water were selected as liquid objects. We use the method of titration to prepare ethylene glycol solutions with different pH values and deionized water with different pH values. Among them, deionized water is used as a reference. Table 1 shows some properties of ethylene glycol and deionized water. 

Solid trichloroacetic acid (TCA) and sodium hydroxide (NaOH) were selected as the acidic and the alkaline substance, respectively, to vary PH values. In the liquid titration process, A pH meter (SG23 SevenGo Duo, Metler-Toledo, Zurich, Switzerland) with a specialist electrode (Inlab 427, Metler-Toledo, Zurich, Switzerland) was used to determine the pH value of aqueous and oil solutions in high precision. The initial pH of ethylene glycol and deionized water was measured to be 7.

First, put the trichloroacetic acid (TCA) crystals in the ethylene glycol at a volume ratio of 1:1000, and perform ultrasonic vibration for 10 min to stir to complete the preparation of the acidic solution required for the titration. Then put sodium hydroxide (NaOH) into ethylene glycol at a volume ratio of 1:1000, and perform ultrasonic vibration for 10 min to stir to complete the alkaline solution required for titration. Then, add the prepared acidic solution and alkaline solution to ethylene glycol by titration. Use a pH meter to obtain an acidic solution of ethylene glycol with a pH in the range of 3–7 and an alkaline solution of ethylene glycol with a pH in the range of 7–11.

Then the prepared acidic solution and alkaline solution are added to deionized water by titration to obtain deionized water with a pH value of 3–11.

The ionic concentration of the experimental liquid used to calculate the Debye length in Equation (13) is obtained by using the simple formula *n* = (*V*_1_*n*_1_ + *V*_2_*n*_2_)/(*V*_1_ + *V*_2_), where *V*_1_ and *n*_1_ were the volume and ionic concentration of ethylene glycol and DI water solution, respectively; *V*_2_ and *n*_2_ were the volume and ionic concentration of TCA and NaOH used to vary the pH values respectively.

#### 2.3.2. Sample of Surfaces

In this experiment, a flat borosilicate gales sample and three samples with different degrees of lipophobicity were prepared, which were nanoparticle composite coatings with superoleophilic, oleophobic, and superoleophobic, respectively. Soda-lime glass with 1.0 mm thickness was used as substrate. 

For the preparation of flat borosilicate glass sample, borosilicate glass samples (CAT. NO. 7101, Sail Brand) were immersed in a piranha solution for 30 min for the removal of surface organic matter. Then, it was rinsed using DI water and ethanol and dried with clean air.

**Figure 3:**
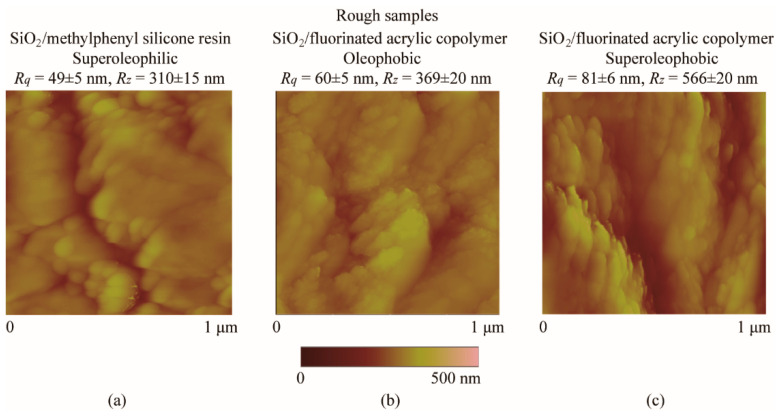
Scanned AFM image in air with measured RSM roughness *R_q_* and P–V distance *R_z_* of (**a**) superoleophilic, (**b**) oleophobic, and (**c**) superoleophobic surfaces.

For the preparation of superoleophilic sample, SiO_2_ nanoparticles with a diameter of 55 ± 15 nm and methylphenyl silicone resin binder were used to form a coating on the flat borosilicate glass substrate via dip-coating method [33]. For the preparation of oleophobic and superoleophobic samples, SiO_2_ nanoparticles with a diameter of 55 ± 15 nm and fluorinated acrylic copolymer binder were used to form a coating on the flat borosilicate glass substrate via spraying method. The nanoparticle-to-binder ratio of the oleophobic surface was 0.3 by weight, for the superoleophobic surfaces it was 0.6 by weight. The increased particle to binder ratio leads to a larger roughness, thus enhance the degree of lipophobicity. Figure 3 shows the scanned AFM images of the five samples in the range of 1 um × 1 um size with the RMS roughness *R_q_* of the samples and the peak to valley distance *R_z_* calculated. 

The 290-F4 contact angle measuring instrument (Rame-Hart Inc., Randolph, NJ, USA) was used to measure the contact angle and contact angle hysteresis of glycol solution and aqueous solution (pH range of 3–11) droplets on the samples. 

Table 2 show that the contact angle (CA) and contact angle hysteresis (CAH) of the ethylene glycol droplets and the aqueous droplets on the samples remained constant with the change of pH value. Therefore, the effect of wettability on the solid–liquid interface can be negligible while the pH value of the solution is changed, and the coupling influence caused by surface wettability is separated.

## 3. Results and Disscusion

### 3.1. Flat Borosilicate Glass Experiments

Experiments were firstly carried out on the flat borosilicate glass to obtain the surface charge density and the slip length of the sphere. Due to the same material, the surface charge densities and slip lengths of the probe and surface are considered equivalent, i.e., *σ*_1_ = *σ*_2_ and *b*_2_ = *b*, respectively. 

When the AFM colloidal probe approaches the flat borosilicate glass sample immersed in ethylene glycol and deionized water with varying pH value, the electrostatic forces applied on the sphere can be obtained. Under the assumption of *σ*_1_ = *σ*_2_, Equation (13) can be used to fit the electrostatic force to obtain the surface charge density of the sphere. As the present method only provides the absolute value of surface charge density, the point of zero charge (PZC) is needed. Previous studies showed that the PZC of glass is about pH = 2–3, so the sphere and borosilicate glass immersed in the solutions with pH = 3 are assumed to be negatively charged. Then the sign of the surface charge density of sphere can be given. The result is shown in Table 3. 

When the AFM colloidal probe approaches the flat borosilicate glass sample immersed in ethylene glycol and deionized water with varying pH value, the hydrodynamic forces applied on the sphere can be obtained. Under the assumption of *b*_2_ = *b*, Equation (2) can be used to fit the hydrodynamic forces to obtain the slip length of the sphere. The result is shown in Table 3. It can be noted that the slip lengths of the sphere immersed in deionized water maintain constant to be ~0, it thus can be ignored while using Equation (14) to fit the hydrodynamic force to obtain the effective slip length of rough surfaces immersed in deionized water.

### 3.2. Rough Surface Experiments

#### 3.2.1. The Measured Electrostatic Force

**Figure 4:**
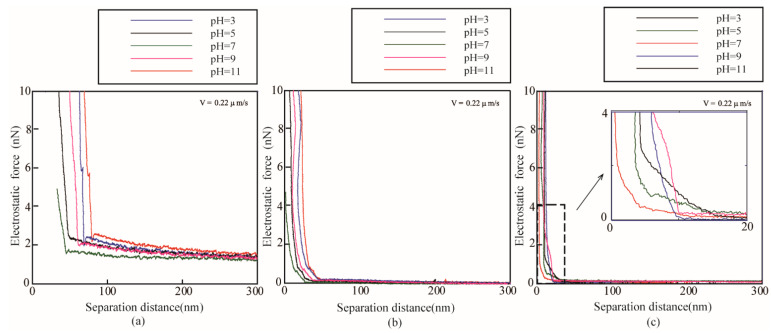
Measured electrostatic force of (**a**) superoleophilic, (**b**) oleophobic, and (**c**) superoleophobic surfaces immersed in ethylene glycol with different pH value obtained at a sphere velocity of 0.22 μm/s.

Figure 4 shows the measured electrostatic forces *F_electro_* applied on the AFM probe approaching the surfaces immersed in ethylene glycol with varying pH values as a function of separation distance *D*. For the oleophilic, oleophobic and superoleophobic surfaces, when the liquid PH value is in the range of 3–7, the electrostatic force is proportional to the liquid PH value. When the liquid PH value is in the range of 7–11, the electrostatic force is inversely proportional to the liquid PH value. There is crossover on the curve of the electrostatic force applied on the colloidal probe because the different ionic concentrations lead to a varying Debye length. The measured electrostatic forces *F_electro_* can be used to obtain surface charge density according to Equation (13).

**Figure 5:**
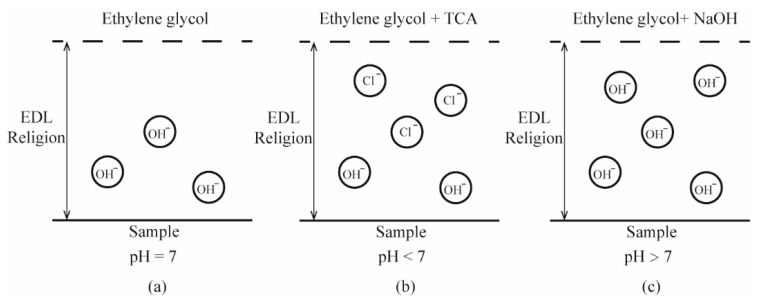
Schematic of the surface charge on the superoleophilic, oleophobic, and superoleophobic surface immersed in ethylene glycol with value of (**a**) pH = 7, (**b**) pH < 7, and (**c**) pH > 8.

This result represents the effect of pH on surface charge density, and it can be explained by the charging mechanism of the sample in the ethylene glycol solution as shown in Figure 5. As a non-electrolyte liquid, the ethylene glycol contains very few ions. When the surface of the sample is immersed in ethylene glycol, due to the electric double layer effect, the surface has a strong adsorption capacity for negative ions. By adsorbing a small amount of hydroxyl ions, it will be charged with a weak negative charge. The increasing ionic concentration can lead to an increase in the capacity of EDL, and thus results in an increase in the magnitude of surface charge density. When the sample surface is immersed in an ethylene glycol acid solution, due to the addition of TCA, the ion concentration of the liquid is significantly increased. When the pH value increases from 3 to 6, the decrease in the magnitude of the surface charge density with decreasing bulk ionic concentration, and the absolute value of surface charge density decreases. In despite the presence of acidoid inhibiting the absorption of hydroxyl ions, it will not affect the adsorbing of Cl^−^ from the decomposition of TCA on the surface. When the sample surface is immersed in an alkaline solution of ethylene glycol, due to the addition of sodium hydroxide, the number of OH^−^ ions adsorbed on the surface increases. When the pH value increases from 6 to 10, both the increasing absorption of hydroxyl ions and the increasing bulk ionic concentration with increasing pH leads to an increase in the final magnitude of surface charge density.

**Figure 6:**
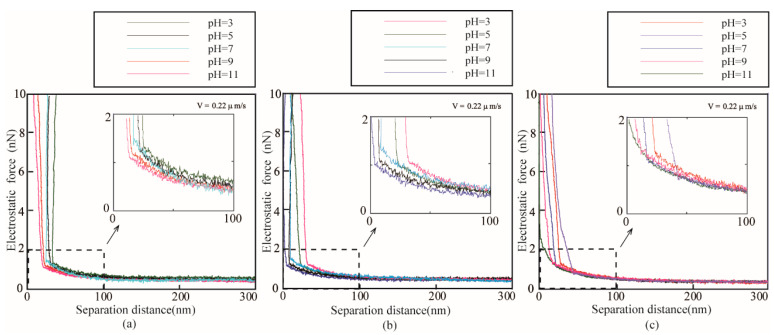
Electrostatic force of (**a**) superoleophilic, (**b**) oleophobic, and (**c**) superoleophobic surfaces immersed in DI water with different pH value obtained at a sphere velocity of 0.22 μm/s.

Figure 6 shows the measured electrostatic forces *F_electro_* applied on the AFM probe approaching the surfaces immersed in DI water with varying pH values as a function of separation distance *D*. For the oleophilic, oleophobic and superoleophobic surfaces, the electrostatic force decreases with the increasing pH value from 3 to 11.

The experimental results of the DI water are different from that of the ethylene glycol solution, which can be explained by the difference in the properties of the two liquids. When the sample surface is immersed in water, due to the dissociation of the silanol groups on the surface, the sample surface adsorbs OH^−^ ions and becomes negatively charged, and the amount of adsorbed OH^−^ ions is much greater than that of ethylene glycol. When the sample surface is immersed in an acidic aqueous solution, the increase in H^+^ ion concentration inhibits the dissociation of silanol groups on the surface, and the OH^−^ ion concentration decreases as the pH value decreases. However, since the coverage of OH^—^ affects the surface adsorption of CCl_3_COO^−^ and Cl^−^ from the decomposition of trichloroacetic acid, the number of adsorbed negative ions is less than the decrease of OH^−^, the absolute value of surface charge density decreases with the decreasing pH value. When the sample surface is immersed in an alkaline aqueous solution, the number of OH^−^ ions adsorbed on the surface increases due to the addition of sodium hydroxide, thereby increasing the ion concentration on the surface, so the absolute value of the surface charge density increases with the increasing pH value.

#### 3.2.2. Measured Hydrodanamic Force

**Figure 7:**
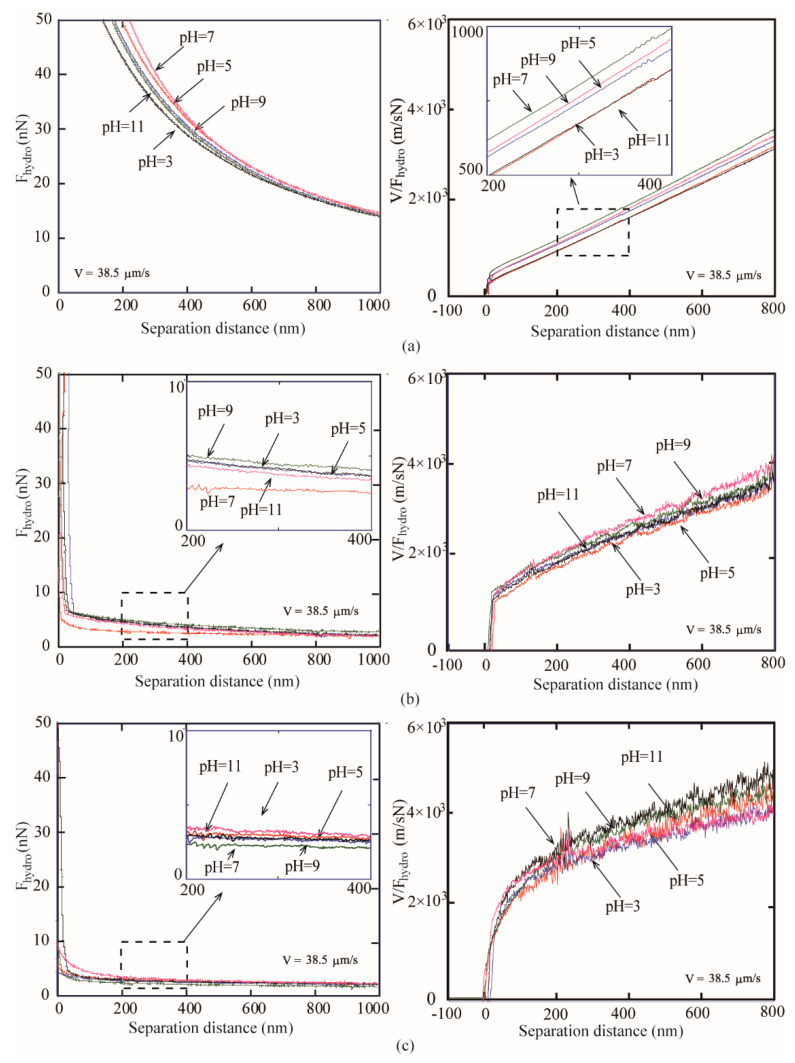
Hydrodynamic force *F_hydro_* and *V/_Fhydro_* of sphere on (**a**) superoleophilic, (**b**) oleophobic, and (**c**) superoleophobic surfaces immersed in DI water with different pH values obtained at a sphere velocity of 38.5 μm/s.

For experiments of samples immersed in ethylene glycol with different pH value, when the colloidal AFM probe approaches to the superoleophilic, oleophobic, and superoleophobic surfaces at a velocity of 38.5 μm/s, the hydrodynamic force *F_hydro_* and *V/F_hydro_* applied on the sphere are shown in Figure 7. Effective slip length is obtained by measuring the hydrodynamic force.

Figure 7 shows the measured hydrodynamic force *F_hydro_* and *V/F_hydro_* as a function of separation distance. It can be found that the results of the experiment on the superoleophilic, oleophobic, and superoleophobic surfaces are similar. In the pH range of 3 to 7, the hydrodynamic force *F_hydro_* applied on the colloidal probe increases with an increasing pH value. The plots of the *V/F_hydro_* curve shifts to the left with the increasing pH value. This means that the interception of the plot on the separation distance shifts to the left and leads to the increase in slip length. In the pH range of 7 to 11, as the pH value increases, the hydrodynamic force *F_hydro_* applied on the colloidal probe decreases with an increasing pH value. The plots of the *V/F_hydro_* curve shifts to the right with the increasing pH value. This means that the interception of the plot on the separation distance shifts to the right and leads to the decrease in slip length. 

For experiments of samples immersed in DI water with different pH value, in the pH range of 3 to 11, the hydrodynamic force *F_hydro_* applied on the colloidal probe increases with an increasing pH value. The plots of the V/Fhydro curve shifts to the left with the increasing pH value. This means that the interception of the plot on the separation distance shifts to the left and leads to the increase in slip length. *V/F_hydro_* can be used to obtain the effective boundary slip length *b_eff_* on the solid–liquid interface with different pH values according to Equation (14).

### 3.3. The Effect of Surface Charge on the Effective Slip Length

**Figure 8:**
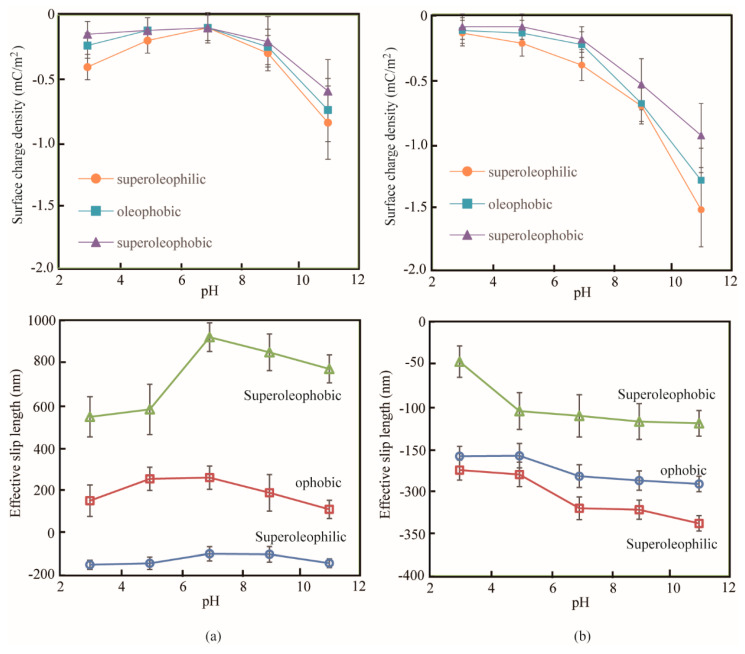
The effect of the surface charge density on the effective boundary slip length of rough samples immersed in (**a**) ethylene glycol and (**b**) DI water with different pH values.

The surface charge density and effective boundary slip length of samples immersed in ethylene glycol and DI water with various pH value are obtained, where the roughness effect is considered, as shown in Figure 8. It can be noted that the effective slip lengths of surfaces immersed in DI water are negative, and the effective slip lengths of superoleopphilic surfaces immersed in Ethylene glycol are negative. The effective slip length of roughness-induced surface is more complicated than that of flat surface, as it is obtained by investigating the velocity of liquid flow at the reference surface. In this article, the position of reference surface within the surface at the height of *Rq* roughness, which has been discussed earlier. This means that the flow velocity at the reference surface is discontinuous due to the rough structure. The negative slip length data do not represent the inexistence of slippage, it means the effective slip length is smaller than that of positive data obtained by using the quantitative method in Equation (14).

For ethylene glycol with different pH, the plots of effective slip length and surface charge density at solid–oil interface show a similar trend. When the pH value in the range of 3 to 7, the effective slip length increases with the increasing surface charge density; when the pH value in the range of 7 to 11, the effective slip length decreases with the decreasing surface charge density. For DI water, similar results can be obtained. When the pH value in the range of 3 to 7, the effective slip length decreases with the decreasing surface charge density. 

It can be explained with the existence of surface charge at the solid–liquid interface can lead to the change electrostatic force, and introduce an interaction. The increasing surface charge density enhances the interaction between the solid surface and the liquid, and decrease the slip length. Therefore, an increasing magnitude of surface charge density leads to a decrease of slip length. 

## 4. Conclusions

The colloidal probe of an atomic force microscope was used to study the effect of surface charge on the effective slip length of the solid–liquid interface. A quantitative study of the relationship between surface charge density and effective slip length is proposed. Experiments were conducted using glycol (liquid with low surface tension) and deionized water on super oleophilic, oleophobic and super oleophobic surfaces with different surface charge densities. Experiments were carried out respectively on two liquids with a pH in the range of 3–11. The potential mechanism of the effect of surface charge density on the effective slip length were discussed and analyzed.

The results of study on the solid–oil interface of this experiment show that, either the acid solution or the alkaline solution of oil will lead to an increasing slip length, which provides a powerful means of drag control of transportation of low-surface tension liquids in the in many applications of micro-/nano-fluidic channels, including micro-/nano- electro-mechanical systems (MEMS/NEMS), micro-/nano- fluidic systems, and confined biological systems, etc.

## Figures and Tables

**Table 1:** Property of liquids used in the experiments [31].

Liquid	Density (g/cm^3^)	Surface Tension (mN/m)	Dynamic Viscosity (mPa s)
DI water	0.9970	71.99	0.980
Ethylene glycol	1.1135	47.7	16.100

**Table 2:** CA, CAH of sample surfaces immersed in DI water and ethylene glycol.

Type of Coating	Composition	DI Water		Ethylene Glycol	
		CA	CAH	CA	CAH
		(deg)	(deg)	(deg)	(deg)
superoleophilic	SiO_2_ and methylphenyl silicone resin	159 ± 5	7 ± 3	64 ± 2	9 ± 2
oleophobic	SiO_2_ and fluorinated acrylic copolymer	160 ± 4	12 ± 3	89 ± 3	22 ± 3
superoleophobic	SiO_2_ and fluorinated acrylic copolymer	162 ± 4	4 ± 1	150 ± 7	8 ± 3

pH had no effect on contact angles.

**Table 3:** Surface charge density and slip length of sphere immersed in ethylene glycol and deionized water at the pH range of 3 to 11.

Liquid	pH = 3	pH = 5	pH = 7	pH = 9	pH = 11
Surface charge density (mC/m^2^)					
Ethylene glycol	−0.625 ± 0.274	−0.254 ± 0.092	−0.104 ± 0.028	−0.446 ± 0.138	−1.096 ± 0.323
Deionized water	−1.047 ± 0.215	−0.438 ± 0.126	−0.217 ± 0.045	−0.565 ± 0.229	−2.143 ± 0.317
Slip length (nm)					
Ethylene glycol	20 ± 5	34 ± 12	35 ± 10	28 ± 14	12 ± 4
Deionized water	~0	~0	~0	~0	~0
